# Susceptibility of Stored-Product Psocids to Aerosol Insecticides

**DOI:** 10.1673/031.012.13901

**Published:** 2012-11-27

**Authors:** George P. Opit, Frank H. Arthur, James E. Throne, Mark E. Payton

**Affiliations:** ^1^ Department of Entomology and Plant Pathology, Oklahoma State University, 127 Noble Research Center, Stillwater, OK 74078-3033, USA; ^2^ USDA-ARS Center for Grain and Animal Health Research, 1515 College Ave., Manhattan, KS 66502, USA; ^3^ Department of Statistics, Oklahoma State University, 301B MSCS Bldg, Stillwater, Oklahoma 74078-1056, USA

**Keywords:** esfenvalerate, methoprene, Psocoptera

## Abstract

The efficacies of commercial methoprene and esfenvalerate aerosols for control of stored-product psocid pests were evaluated in simulated field studies. The efficacies of methoprene, esfenvalerate EC, the carrier Isopar-M™, and a combination of methoprene and esfenvalerate aerosols for control of *Liposcelis decolor* (Pearman) (Psocoptera: Liposcelididae) and *Liposcelis entomophila* (Enderlein) nymphs were assessed, and the effects of direct and indirect exposure of *Liposcelis bostrychophila* Badonnel, *L. decolor*, and *Liposcelis paeta* Pearman adults to esfenvalerate EC aerosol were evaluated. The greatest nymphal mortality attained was 76%, indicating that the four aerosols tested were ineffective against *L. decolor* and *L. entomophila* nymphs. In the direct and indirect exposure studies, the greatest adult mortalities attained for the three psocid species were 62 and 32%, respectively. Based on these data, esfenvalerate aerosol is ineffective for control of *L. bostrychophila, L. decolor, L. entomophila*, and *L. paeta* psocid species. This study shows that methoprene, esfenvalerate EC, and a combination of methoprene and esfenvalerate aerosols were ineffective against the four psocid species tested when applied at rates that are usually effective against other stored-product insect pests.

## Introduction

Psocids (Psocoptera) are small, soft-bodied insects and are now considered significant pests of stored products (Phillips and Throne 2010). They can persist on a variety of foods, and there is variation in biology and ecology among the different species (Opit and Throne 2008a,b; Opit et al. 2009a,b). The pest status of stored-product psocids is due to the weight losses caused by consumption of germ and endosperm (McFarlane 1982; Kuîerová 2002), their ability to spread fungal pathogens, thereby making them a human health threat (Obr 1978; Kalinovic et al. 2006), allergic responses they cause in sensitized people (Turner et al. 1996), frequent failure of standard practices of protection and disinfestation to control psocids (Wang et al. 1999; Beckett and Morton 2003; Nayak et al. 2003; Nayak and Daglish 2007), and the fact that commodities infested by psocids can be rejected for export (Kučerová 2002; Nayak 2006).

Recent studies have shown psocids to be quite tolerant to some of the currently used insecticides when applied at rates usually effective for control of other stored-product insect pests. Previously, it was generally assumed that control measures targeted at other stored-product insects, such as Coleoptera and Lepidoptera, also controlled psocids. However, this has been proven untrue. For example, the stored-product psocids *Liposcelis paeta* Pearman (Psocoptera: Liposcelididae) and *Liposcelis entomophila* (Enderlein) are tolerant to fenitrothion, chlorpyrifos-methyl, pirimiphosmethyl, deltamethrin, bioresmethrin, and carbaryl (Nayak et al. 1998). The psocids *L. entomophila, Liposcelis decolor* (Pearman), and *Lepinotus reticulatus* Enderlein have been shown to be less susceptible than many other stored-product insects to three different types of diatomaceaous earths, namely, Dryacide®, Protect-It®, and Insecto®, applied to wheat, rice, and maize, with mortalities after two weeks of exposure barely exceeding 70% (Athanassiou et al. 2009). *Liposcelis* spp. are also tolerant to permethrin and pirimiphosmethyl applied as structural treatments (Collins et al. 2000; Nayak et al. 2002) and to spinosad applied as a grain protectant (Nayak and Daglish 2007). However, chlorfenapyr and beta-cyfluthrin applied on concrete surfaces are effective against *L. entomophila* and *L. reticulatus*, whereas Pyrethrins are not effective (Guedes et al. 2008).

Aerosol treatments are being used more frequently in warehouses and flour mills to reduce the need for fumigants for stored-product insect control. During an aerosol treatment, a volume of space is treated with a relatively small amount of usually nonresidual insecticide, with droplets having a diameter of ∼20 microns or less, in order to control the exposed stages of flying and crawling insects. Aerosols can be an effective technique for controlling stored-product moths and beetles in complex environments, such as warehouses or flour mills. However, there are currently no published studies on the use of aerosols for the control of psocids of the genus *Liposcelis*, which represent the vast majority of the stored-product psocid pest species. The only published aerosol treatment study on psocids involved *Lepinotus patruelis* Pearman (Pinniger 1985). Aerosol applications in that study, which was conducted in a large wholesale food warehouse using application rates of 3 and 33 mg/m^3^ of an experimental formulation of permethrin applied using a CO2 propellant system, resulted in 99 and 100% psocid mortality, respectively. Field tests with aerosol insecticides using either immature or adult *Tribolium* spp. have involved either direct exposure of immature and adult insects (Arthur 2008; Arthur and Campbell 2008) or an indirect method, whereby a concrete substrate was exposed to the aerosol, and then immature and adult beetles were placed on the substrate (Arthur 2010). The insect growth regulators (IGRs) methoprene and fenoxycarb have been shown to have ovicidal effects and to drastically reduce the population growth and fertility of *Liposcelis bostrychophila* Badonnel when added to psocid diet (Buchi 1994). Therefore, given the potential of IGRs and pyrethroids to cause high psocid mortality, the objectives of this study were to evaluate commercial pyrethroid and IGR aerosols for their efficacy against psocids of the genus *Liposcelis* using direct and indirect exposure methods, and to examine variation in the response of selected psocid species to these aerosols.

## Materials and Methods

### Nymphal Mortality Study

The efficacies of methoprene (Diacon II®, 288 mg/mL active ingredient (AI) Emulsifiable Concentrate (EC), obtained from Central Sciences International, http://www.centrallifesciences.com/), esfenvalerate (Conquer®, 3.48% AI, or approximately 30 mg AI per mL, obtained from MGK Corporation, http://www.mgk.com/), the carrier Isopar-M™ used to formulate the aerosols (Exxon Mobil Chemical Company, http://www.exxonmobilchemical.com/), and a combination of methoprene and esfenvalerate applied as aerosols for control of *L. entomophila* and *L. decolor* first (N1) and second (N2) instars were evaluated. Only nymphs were used in this study because IGRs generally are targeted toward immature insects. The two instars were determined based on their size and color—they are usually the smallest and most light-colored nymphs among insects obtained from culture jars containing individuals of variable age. N1 and N2 were obtained by spreading a portion of the contents of a jar containing cracked wheat diet used for rearing psocids on a 9-cm Petri dish, which had a coat of Fluon® (Sigma-Aldrich, http://www.sigmaaldrich.com/) on the inner walls, and removing the nymphs using a moist brush under a stereomicroscope (Zeiss Stemi 2000-C, http://www.zeiss.com/). Cultures of *L. entomophila* and *L. decolor* used in the study were started with insects collected during the summer in 2004 and 2006, respectively, from wheat stored in steel bins at the Center for Grain and Animal Health Research in Manhattan, KS. Voucher specimens of *L. decolor* and *L. entomophila* used in this study were deposited in the Kansas State University Museum of Entomological and Prairie Arthropod Research under Lot Numbers 182 and 205, respectively. Both psocid species were reared on a mixture of 97% cracked hard red winter wheat, 2% Rice Krispies (Kellogg USA Inc., http://www.kelloggs.com/), and 1% brewer's yeast (MP Biomedicals, http://www.mpbio.com/) (wt:wt; cracked wheat diet) in 0.473-liter glass canning jars covered with mite-proof lids. Cultures were maintained at 30° C, 75% RH, and 24 hour scotophase.

Field trials were conducted in five outdoor sheds that have been used for a number of small-scale aerosol trials. Complete descriptions of the interiors of these sheds have been given in previous publications (Toews et al. 2005; Jenson et al. 2009, 2010). The interior of each of the sheds used in the tests was 32.2 m^3^. Three metal shelves approximately 0.15 m in height were placed on the floor inside each shed. These shelves were about 0.6 m away from the wall and centered on the back wall (east) and the north and south sides. Individual arenas were created using the bottom of a plastic Petri dish that had a measured inside area of 62 cm^2^. The side was 2.3 cm high. A dry driveway patching material (Rockite®) was mixed with water to form a slurry, and poured into each of thirty Petri dishes to an approximate depth of 0.5 cm. A thin coating of Fluon® was applied to the interior wall of each arena using a small paintbrush in order to prevent psocids escaping from the arenas. Twenty N1 and N2 psocids were placed in each arena. A replicate for each species consisted of three arenas placed on the same shelf of each of the five sheds, i.e., each replicate contained three subsamples. The four treatment aerosols were applied in four of the sheds. The fifth shed served as the untreated control.

Aerosols were applied using a hand-held ultralow volume applicator (model no. E2 MLD® Chemical Dispersal Unit, MicroGen Equipment Corporation, http://pestcontrol.basf.us/). Production and manufacturing of this unit has ceased, so it is not available for purchase. The esfenvalerate was diluted with the carrier Isopar-M™ in accordance with label directions, which specify mixing 1 ounce or about 30 mL of the esfenvalerate in 3,784 mL of the Isopar-M™ carrier, and then spraying the diluted solution at the rate of 30 mL/28.3 m^3^ of airspace volume inside the shed (36 mL total, or 0.26 mg AI/m^3^ in each shed). The aerosol was applied by standing about 3 m inside the doorway with the door closed, pointing the nozzle of the ultralow volume applicator to the back of the shed, and slowly pivoting the applicator as the spray was dispensed.

Solutions were weighed before and after application to ensure that the approximate target amount of chemical (AI/m^3^) was applied to each shed. Label directions for methoprene specify 3 mL of the EC in 3,784 mL of the oil carrier/280 m^3^ of headspace (3.08 mg AI/m^3^). The methoprene EC was added to the mixture of esfenvalerate and Isopar-M™ to obtain the proper ratio.

The shed remained closed for two hours after application of the aerosol, in accordance with label directions, after which the arenas in each shed were removed and returned to an indoor laboratory at the Center for Grain and Animal Health Research, Manhattan, KS, to count and remove dead psocids immediately. Approximately 5 g of cracked wheat was added to each arena, and all arenas were placed in an incubator set at 27° C and 60% relative humidity. After two weeks, the number of dead psocids (incapable of moving when prodded) in each arena was again determined under a stereomicroscope. The experiment consisted of two replications over time.

Analysis of variance procedures (PROC MIXED) were used to determine the effect of species, treatment (type of aerosol), and time elapsed after treatment (zero or two weeks) on mortality (SAS Institute 2007). The arcsine square root transformation was applied because the response variable was percentage data, which tend to not be normally distributed and to have non-homogeneous variances (Sokal and Rohlf 1981). Untransformed means and standard errors are reported to simplify interpretation. A randomized complete block model with repeated measures and an autoregressive (period one) covariance structure was used to model the data. Least squares means within treatments were compared with pairwise ttests when simple effects were significant. A 0.05 level of significance was used for all comparisons.

### Adult Mortality Studies

Materials and methods were similar to those described above for the nymphal aerosol study except the field trials were conducted in only two of the five outdoor sheds and the psocid species *L. bostrychophila, L. decolor*, and *L. paeta* were used. Other differences were that only esfenvalerate was tested, and the effects of direct and indirect exposure of adults to the insecticide were also tested. Cultures of *L. bostrychophila* and *L. paeta* used in the study were started with insects collected during the summer of 2006 from a grain elevator at the Center for Grain and Animal Health Research in Manhattan, KS. Voucher specimens of *L. bostrychophila* and *L. paeta* used in this study were deposited in the Kansas State University Museum of Entomological and Prairie Arthropod Research under Lot Numbers 202 and 207, respectively. The source of *L. decolor* was the same as in the nymphal mortality study. All psocid species were reared as already described (Opit and Throne 2008a).

For each species, a replicate for the direct exposure study consisted of 20 adults of variable age placed in each of two arenas assigned to that species. One set of six arenas, two for each species, was placed on the shelf along the south wall of one of the sheds described above, i.e., each replicate contained two sub-samples. A replicate for the indirect exposure was similar except there were no psocids in arenas; these arenas were placed on the same shelf as those for direct exposure. This same arrangement was followed for a matching set of arenas placed in a second shed not treated with the aerosol (the untreated control).

Two hours after application of the aerosol, the arenas containing the psocids were examined, and individuals were classified as having survived the aerosol (actively running), knocked down (on their backs and moving), or dead (incapable of moving when prodded). Approximately 5 g of cracked wheat was added to each arena, and all arenas were placed in an incubator set at 27° C and 60% relative humidity. Each of the arenas was removed after 48 hours, and psocids were counted and classified as described above. The arenas from the untreated control shed were processed in the same manner. For the indirect exposures, 20 adults of *L. bostrychophila, L. decolor*, or *L. paeta* and 5 g of cracked wheat were placed in each of two arenas removed from the shed treated with the aerosol, and the same procedure was followed for arenas removed from the untreated shed. All of these arenas were placed in the incubator. The following day, the arenas were removed from the incubator, and psocids were classified as having survived, knocked down, or dead. The arenas then were returned to the incubator and examined daily for six days.

Six separate replications were conducted in the summer of 2008 and six were conducted in the summer of 2009. These two sets of six replications were considered to be two blocks. All raw data for each of the two methods of exposure (direct and indirect) were converted to percentage values. Data analysis for the direct exposure study of adults was conducted using PROC MIXED in SAS to determine the effects of treatment (versus control) and time after treatment (zero or two days) on mortality (SAS Institute 2007). Mortality was the only variable analyzed because knockdown ranged from only 0 to 6.3%. Data were transformed by arcsine square root as described above, and a randomized complete block model with repeated measures and an autoregressive (period one) covariance structure was used to model the data. Untransformed means and standard errors are reported to simplify interpretation. Simple effects and mean comparisons were as described above. Data analysis for the indirect exposure study was also conducted as described above, with the addition of time (days after treatment) as a repeated measure, using an autoregressive correlation structure to model the within subject correlation. Knockdown ranged from 0 to 6.7% for all three species; hence, mortality was the only variable analyzed for this dataset as well.

## Results

### Nymphal Mortality Study

Percentage mortality differed among types of aerosol treatments (*F* = 9.7; df = 4,8.9; *p* = 0.003) and time elapsed after treatment (*F* = 111.2; df = 1,87.9; *p* < 0.001), and treatment by time interaction was significant (*F* = 9.1; df = 4,87.9; *p* < 0.001). Percentage mortality did not differ with species (*F* = 0.8; df = 1,8.9; *p* = 0.404) and none of the interaction terms that included species were significant (species * treatment: *F* = 0.9, df = 4,8.9, *p* = 0.522; species * time: *F* = 0.1, df = 1,87.9, *p* = 0.728; species * treatment * time: *F* = 0.4; df = 4,87.9; *p* = 0.839). Despite lack of species differences, data were then analyzed by species with 2-way ANOVA's to enable the comparison of data for each species with previous studies with other insecticides. The patterns for mortality of *L. decolor* for the five treatments varied at zero and two weeks after treatment application, as indicated by a significant test for interaction (*F* = 3.1; df = 4,42.9 ; *p* = 0.025), so 1-way ANOVA's were used to test for differences among aerosol treatments within a time period. Mortality differed among the treatments at both zero and two weeks after treatments were applied (zero weeks: *F* = 3.2; df = 4,23; *p* = 0.031; two weeks: *F* = 7.4; df = 4,23; *p* < 0.001; Table 1). At week zero, esfenvalerate and Isopar-M caused significantly higher mortality than in the control, but mortality did not differ among the four aerosol treatments. At week two, esfenvalerate, the combination of methoprene and esfenvalerate, and Isopar-M™ caused significantly higher mortality than in the control, while mortality caused by methoprene was similar to that in the control and IsoparM™ treatments. The highest mean mortality was only 63%, and this mortality was achieved two weeks after esfenvalerate application.

Similarly, the patterns for mortality of *L. entomophila* for the five treatments varied at zero and two weeks after treatment application, as indicated by a significant test for interaction (*F* = 7.2; df = 4,45; *p* < 0.001). Mortality did not differ among the treatments zero weeks after the treatments were applied (*F* = 2.1; df = 4,24; *p* = 0.117; Table 1). However, mortality differed among the treatments two weeks after treatments were applied (*F* = 28.5; df = 4,24; *p* < 0.001), with the combination of methoprene and esfenvalerate, esfenvalerate alone, and IsoparM™ causing significantly higher mortality than in the control; mortality caused by methoprene was similar to that in the control and Isopar-M™ treatments. The highest mean mortality was only 76%, and this mortality was reached two weeks after application of a combination methoprene and esfenvalerate.

### Adult Mortality Studies

Percentage mortality in the direct exposure study differed between the aerosol treatment and the untreated controls (*F* = 34.3; df = 1,25; *p* < 0.001), but did not vary with species (*F* = 0.4; df = 2,25; *p* = 0.681) or time (*F* = 2.9; df= 1,102; *p* = 0.089). No interactions were significant. Mortality in untreated controls ranged from 0 to 7%, and no untreated psocids were knocked down but not dead. Mortality of psocids exposed directly to the esfenvalerate aerosol ranged from 46 to 59% and 45 to 62% immediately (zero) and two days after exposure, respectively, and always was greater than the corresponding control mortality (Table 2). Although mortality in treatments was always greater than in the control, mortality was always less than 62%.

Percentage mortality in the indirect exposure study differed between the treatment and controls (*F* = 33.6; df = 1,63.1; *p* < 0.001) and among the one to six day post-exposure observations (*F* = 14.2; df = 5,307; *p* < 0.001), but did not differ with species (*F* = 0.61; df = 2,63.1; *p* = 0.549). Neither the species by treatment nor the treatment by exposure time interactions were significant when analyzed with time as a repeated measure. Occasionally, the value recorded for mortality on a particular day for one of the three psocid species was slightly lower than the previous day, because of the difficulty of determining whether the psocids were dead due to their small size. To examine the differences in mortality between treatments and controls at the different post-exposure times and to compare data for each species with previous studies with other insecticides, data were analyzed by species, even though species was not significant in the overall analysis. Mortality of all species generally increased each day in the aerosol treatments (Tables 3, 4, and 5 for *L. bostrychophila, L. decolor*, and *L. paeta*, respectively) and was significantly greater than in the untreated controls from days three to six for *L. bostrychophila* and *L. decolor*, and days four to six for *L. paeta*. Mortality was always less than 33% for all species, indicating that this particular insecticide was not effective, or that the psocids are not picking up enough of the aerosol from the treated surface.

## Discussion

Esfenvalerate, methoprene, and a combination of methoprene and esfenvalerate aerosols were not effective against *L. decolor* and *L. entomophila* nymphs. The greatest mortality two weeks after treatment with methoprene aerosol was only ∼16%. Although esfenvalerate and a combination of esfenvalerate and methoprene resulted in much greater mortality of *L. decolor* and *L. entomophila* nymphs two weeks after aerosol application, the mortalities attained were not great enough for commercial use and indicate poor direct efficacy of these two treatments toward psocid nymphs. When *L. decolor* nymphs were treated with esfenvalerate or the combination treatment, mortalities of 63 and 51%, respectively, were the greatest attained; for *L. entomophila*, mortalities of 58 and 76%, respectively, were attained. Immediately (zero weeks) after aerosol application on *L. decolor* nymphs, esfenvalerate and the carrier caused significantly higher mortality than in the control; however, none of the treatments resulted in significantly greater mortality than in the control for *L. entomophila* at zero weeks. This may suggest that *L. decolor* is slightly more susceptible to esfenvalerate aerosol than *L. entomophila*. Two weeks after aerosol application, the carrier Isopar-M™ resulted in significantly greater mortality than the control for both psocid species. This indicates that a significant part of the mortality caused by esfenvalerate and the combination of methoprene and esfenvalerate may be due to Isopar-M™. Methoprene still had no significant effect on psocid mortality even two weeks after aerosol application.

IGRs can exhibit potent insecticidal activity (Cutler et al. 2006) and they typically kill juvenile insects within three to 10 days, depending on the product.

Esfenvalerate and a combination of methoprene and esfenvalerate significantly increased psocid mortality two weeks after aerosol application. Similar results were found by Turner et al. (1991) in a study involving five synthetic pyrethroids applied as surface treatments on plywood and glass. In the present study, the increased mortality with time is probably due to psocids picking up more insecticide from the cement arenas as they walk around. The fact that esfenvalerate and a combination of methoprene and esfenvalerate significantly increased psocid mortality two weeks after aerosol application while methoprene did not suggests that it's mostly esfenvalerate and the carrier IsoparM™ that are contributing to psocid mortality in this combination. Therefore, there appears to be no advantage in using the combination treatment for control of nymphs over using esfenvalerate alone. Data from a study that was recently published on effects of methoprene on psocids (Athanassiou et al. 2010) indicated that methoprene applied to stored grains is also ineffective.

Direct exposure of *L. bostrychophila, L. decolor*, and *L. paeta* adults to esfenvalerate aerosol showed that this insecticide applied as an aerosol is ineffective against adult psocids as well. The greatest mortalities achieved for *L. bostrychophila, L. decolor*, and *L. paeta* were 62, 46, and 46%, respectively. Again, these mortalities are not great enough for commercial use and indicate poor direct efficacy of esfenvalerate toward adult psocids. In the study with nymphs, mortality of *L. decolor* two weeks after aerosol application was 63%, but it was 45% two days after direct aerosol application to adults. Much higher levels of psocid mortality were found in a study on the efficacy of permethrin space treatments against *L. patruelis* (Pinniger 1985). That study was conducted in a large wholesale food warehouse using doses of 3 and 33 mg/m^3^ of an experimental formulation of permethrin applied using a CO2 propellant system. The mortalities for the two doses for exposed psocids were 99 and 100%, respectively; the mortalities for protected psocids were 77 and 92%, respectively. The possible reason why these results differed from the results in the present study is that the psocid species, insecticide, and method of aerosol deployment used by Pinniger (1985) were different.

In the indirect exposure study with adults, the greatest mortality achieved in the esfenvalerate aerosol treatment for all species and post-exposure periods (one to six days) was ∼30%. From the two direct exposure studies already discussed, direct exposure of psocids to esfenvalerate aerosol results in increased mortality compared to indirect exposure. Other studies where psocids have been indirectly exposed to pyrethroids have shown that use of these insecticides results in unsatisfactory levels of mortality (Pinniger 1985; Turner et al. 1991). It is likely that the low efficacy of esfenvalerate applied indirectly or directly is because it is not readily penetrating the cuticle.

Contact insecticides and aerosols are often used to control stored-product insect pests inside interior structures such as flour mills, food processing plants, and food warehouses (Toews et al. 2005; Arthur and Peckman 2006). Recent simulated field trials and tests conducted in actual commercial facilities showed that Pyrethrin or pyrethroid aerosols can be effective against adult *Tribolium castaneum*, the red flour beetle, and adult *Tribolium confusum*, the confused flour beetle (Arthur 2008; Arthur and Campbell 2008). However, application of the pyrethroid esfenvalerate at the label rate to wanderingphase larvae of *Plodia interpunctella*, the Indianmeal moth, resulted in only about 7% mortality (Jenson et al. 2010). Similarly, results of the present study indicate that esfenvalerate aerosol is ineffective against *L. bostrychophila, L. decolor, L. entomophila*, and *L. paeta*. Hence, there can be considerable variation among insect species and even life stages in their response to a particular insecticide.

The IGRs methoprene and fenoxycarb have been shown to have ovicidal effects and to drastically reduce the population growth and fertility of *L. bostrychophila* (Buchi 1994). In that investigation of these insecticides, psocids were held on food treated with these two IGRs. That study showed that the reduction in psocid numbers after four and six weeks was 94%, and the conclusion was that the two IGRs had potential for the control of stored-product psocids. However, in the present study where methoprene was directly applied as an aerosol and food was added after the treatment, methoprene was found to be ineffective against *L. decolor* and *L. entomophila*. Results of these two studies suggest that IGRs may be more effective against psocids when ingested with food than when they get into the insect through the cuticle, i.e., direct or indirect exposure, as was the case in the present study.

The effectiveness of esfenvalerate aerosol treatment targeted at psocids might be improved by prior exposure of psocids to a synergist such as piperonyl butoxide (Turner et al. 1991). The use of piperonyl butoxide to synergize permethrin significantly increased the mortality of *L. bostrychophila* to ∼95% (Turner et al. 1991). Based on these data, it appears that only synergized pyrethroid formulations should be used for psocid control and not formulations containing pyrethroids alone (Turner 1994). The use of nonsynergized pyrethroids poses the risk of development of tolerant populations, which would jeopardize continued pyrethroid use (Turner 1994). This study has shown that esfenvalerate aerosol is ineffective against *L. bostrychophila, L. decolor, L. entomophila*, and *L. paeta* nymphs and adults. In addition, methoprene and a combination of methoprene and esfenvalerate are ineffective against *L. decolor* and *L. entomophila* nymphs. It is likely that aerosols of these insecticides are ineffective against other stored-product psocid pests as well. This study again shows that psocids are quite tolerant to many currently used insecticides when applied at rates that are usually effective against other stored-product insect pests.

**Table 1.**
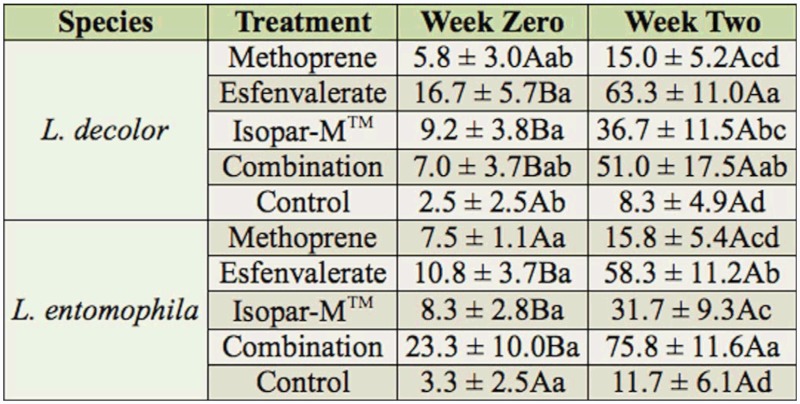
Percentage mortality (mean ± SEM) of *Liposcelis decolor* and *Liposcelis entomophila* first (N1) and second (N2) instars zero and two weeks after direct exposure to aerosol applications of methoprene, esfenvalerate EC, the Isopar-M™ carrier, a combination of methoprene and esfenvalerate, and the control. Means were compared using pairwise t-tests. Means for a treatment within a row followed by different uppercase letters are significantly different, whereas means for a species within a column followed by different lowercase letters are significantly different (*p* > 0.05).

**Table 2.**
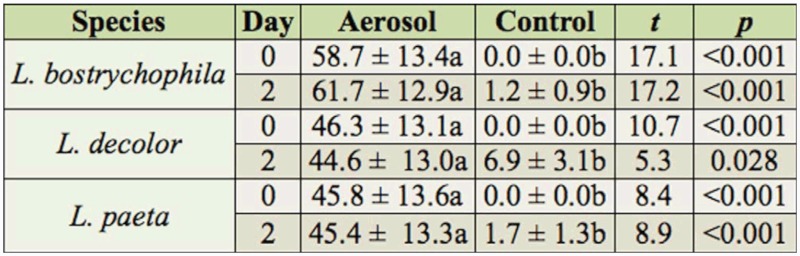
Percentage mortality (mean ± SEM) of *Liposcelis bostrychophila, Liposcelis decolor*, *and Liposcelis paeta* zero and two days after direct exposure to an aerosol application of esfenvalerate and in the untreated control. Means within a row followed by different letters are significantly different (pairwise t-test, Proc Mixed in SAS, df was 1, 27.9 for all comparisons).

**Table 3.**
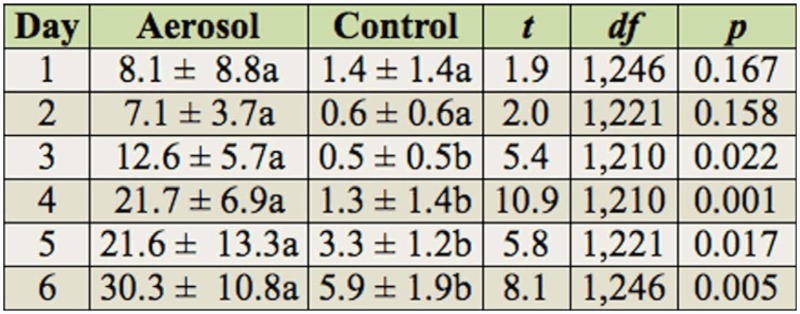
Percentage mortality (mean ± SEM) of *Liposcelis bostrychophila* after one to six days of exposure on untreated concrete arenas and arenas exposed to esfenvalerate aerosol. Means within a row followed by different letters are significantly different (pairwise t-test, Proc Mixed in SAS).

**Table 4.**
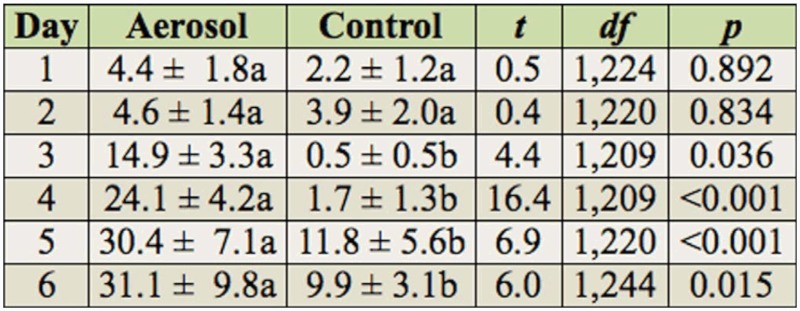
Percentage mortality (mean ± SEM) of *Liposcelis decolor* after one to six days of exposure on untreated concrete arenas and arenas exposed to esfenvalerate aerosol. Means within a row followed by different letters are significantly different (pairwise t-test, Proc Mixed in SAS).

**Table 5.**
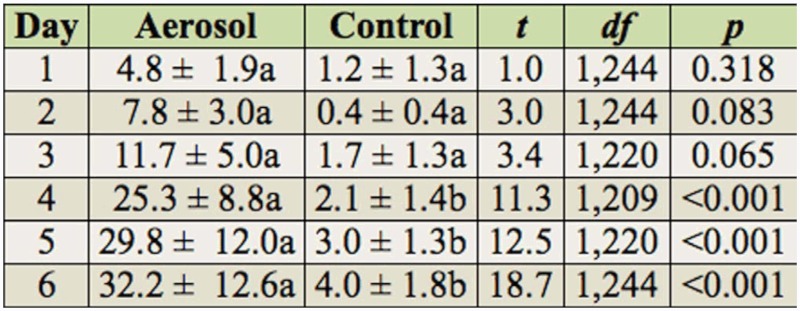
Percentage mortality (mean ± SEM) of *Liposcelis paeta* after one to six days of exposure on untreated concrete arenas and arenas exposed to esfenvalerate aerosol. Means within a row followed by different letters are significantly different (pairwise t-test, Proc Mixed in SAS).

## Abbreviations

AI: active ingredient
IGRs: insect growth regulators
